# Insights in Cell Biomechanics through Atomic Force Microscopy

**DOI:** 10.3390/ma16082980

**Published:** 2023-04-09

**Authors:** Sajedeh Kerdegari, Paolo Canepa, Davide Odino, Reinier Oropesa-Nuñez, Annalisa Relini, Ornella Cavalleri, Claudio Canale

**Affiliations:** 1 Dipartimento di Fisica, Università di Genova, Via Dodecaneso 33, 16146 Genova, Italy; sajedeh.kerdegari@edu.unige.it (S.K.); paolocanepa@unige.it (P.C.); odino@fisica.unige.it (D.O.); relini@fisica.unige.it (A.R.); 2 Department of Materials Science and Engineering, Uppsala University, Ångströmlaboratoriet, Box 35, SE-751 03 Uppsala, Sweden; reinier.oropesa@angstrom.uu.se

**Keywords:** AFM, cells, mechanobiology, cell mechanical properties

## Abstract

We review the advances obtained by using Atomic Force Microscopy (AFM)-based approaches in the field of cell/tissue mechanics and adhesion, comparing the solutions proposed and critically discussing them. AFM offers a wide range of detectable forces with a high force sensitivity, thus allowing a broad class of biological issues to be addressed. Furthermore, it allows for the accurate control of the probe position during the experiments, providing spatially resolved mechanical maps of the biological samples with subcellular resolution. Nowadays, mechanobiology is recognized as a subject of great relevance in biotechnological and biomedical fields. Focusing on the past decade, we discuss the intriguing issues of cellular mechanosensing, i.e., how cells sense and adapt to their mechanical environment. Next, we examine the relationship between cell mechanical properties and pathological states, focusing on cancer and neurodegenerative diseases. We show how AFM has contributed to the characterization of pathological mechanisms and discuss its role in the development of a new class of diagnostic tools that consider cell mechanics as new tumor biomarkers. Finally, we describe the unique ability of AFM to study cell adhesion, working quantitatively and at the single-cell level. Again, we relate cell adhesion experiments to the study of mechanisms directly or secondarily involved in pathologies.

## 1. Introduction

The introduction of the Atomic Force Microscope (AFM) in 1986 [1] paved the way for the real-space, high-resolution analysis of soft/biological samples in hydrated/physiological conditions [2,3,4,5,6].

The core of an AFM is a soft cantilever with a tip at its apex which is brought in close proximity to the sample surface; the interaction forces between the tip and the sample induce a deflection of the cantilever. The sample morphology can be reconstructed by monitoring the cantilever deflection while the tip is scanning over the sample.

The AFM demonstrated its ability to work on planar systems, which are particularly suitable to exploit the high resolution inherently related to the technique. Planar systems are generally model systems. They are important tools for the study of molecular interactions on a simplified subgroup of molecules that are part of the “real” biological system. The AFM has been employed in the characterization of very thin molecular films, such as self-assembled monolayers [7,8,9,10,11,12,13], Langmuir–Blodgett films [14,15,16,17,18], supported lipid bilayers (SLBs) [19,20,21], and protein layers [2,22,23].

Although initially introduced as an imaging tool, it was quickly clear that the use of the AFM could go beyond this application. The capability to investigate the interaction between tip and sample with very high sensitivity, together with the precise control of the lateral tip position, enabled the probing of the sample with a lateral resolution at the nanometer scale and with a force sensitivity in the pN range [2,24,25].

The applications of the AFM as a spectroscopic tool have developed rapidly over recent years, focusing particularly on the characterization of soft matter or extremely thin samples, where standard indentation methods are severely limited. In material sciences, the AFM became a standard tool for the morpho-mechanical characterization of soft polymers. Particularly interesting are the applications on nanocomposite materials [26], where the AFM can discriminate between the elastic modulus of the polymer matrix and that of the nanoparticle reinforcement [27,28].

However, the improvement of AFM as a force spectroscopy tool has found its most significant applications in biomechanics, for the study of complex biological samples, such as cells and tissues. These large, highly rough, and generally poorly adherent samples frustrated the AFM’s imaging capabilities. It was clear from the beginning that the use of the AFM in force spectroscopy mode could be more informative. The high sensitivity of force spectroscopy was successfully exploited to monitor the nanomechanical vibration of living cells, from bacteria [29] to oocytes [30]. The AFM-derived vibrational profiles of neuronal cells and tissues allowed for the real-time discrimination between healthy and malignant brain tissues [31].

A broad field of application of force spectroscopy focused on the determination of the elastic properties of cells and tissue [32,33,34]. Nowadays, several works demonstrated that changes in cell stiffness are associated with pathologies [35,36]. These works opened the way for extending the application of AFM to medicine, where the AFM is not only used as a tool for defining the molecular mechanisms at the base of pathological states, but also as a diagnostic tool.

AFM force spectroscopy has been applied not only in nanoindentation experiments but also as a quantitative method to study cell adhesion, by monitoring the cantilever deflection during a cell detachment process. Cell adhesion has a primary role in the regulation of the cell cycle in multicellular organisms and is also involved in the development of diverse pathological states [37,38].

Despite the recognized biological significance, cell adhesion properties are often determined on the basis of qualitative essays, such as the washing assay that was introduced several decades ago [39,40]. Other approaches based on the application of shear forces have been proposed more recently. Thickness Shear Mode (TSM) sensors as well as Quartz Crystal Microbalances (QCMs) are used to obtain an indirect evaluation of cell adhesion and the kinetics of the process [41,42]. Flow cytometric assay has been employed to estimate the adhesion of human colon adenocarcinoma cells on endothelial cells, and the adhesion of monocytes on endothelial cells in atherosclerosis [43]. All these techniques only provide a qualitative analysis of cellular adhesion strengths. Furthermore, they provide results averaged from the entire cell population, losing information about cell-to-cell differences.

Single-cell force spectroscopy (SCFS) offers the unique capability to quantify cell adhesion properties at the level of an individual cell, hence discriminating the subtle variations in adhesion between single cells or between the cell and a molecular substrate, which may have biological significance. SCFS experiments can also be performed by using a pipette-based technique to capture the cell and measure cell adhesion with a biomembrane force probe apparatus [44] or an optical tweezer [45]. The main advantage of these two approaches is related to their high sensitivity, being able to detect forces between 0.2 pN and 200 pN; however, the AFM offers a wider range of detectable forces, from 10 pN to 100 nN, allowing a broader set of biological issues to be addressed. Furthermore, the AFM offers accurate control of the cell position along the three spatial axes during the experiments. All the techniques described above have been exploited to access biological mechanisms hitherto inaccessible to the classic approaches.

In this work, we will review the advances obtained by using AFM-based approaches in the field of cell/tissue mechanics and adhesion, comparing the solutions proposed, and critically discussing them.

## 2. AFM Nanoindentation Experiments Provide New Insights in Mechanosensing: Are Cells Adapting Their Phenotypes to Microenvironmental Mechanical Properties?

The first AFM data on cell stiffness were presented more than 30 years ago. Initially, all the results were obtained from cells grown on standard substrates for cell culture, such as glass or polystyrene [35,46,47], i.e., extremely rigid substrates when compared with the physiological environment that hosts the cells in a living organism.

Although the experiments on rigid substrates demonstrated that the cell mechanical properties depend on the specific cell type [48], an intriguing question was still open: how do the cells populating different organs and tissues sense, adapt, and react to the mechanical environment in which they are embedded? In particular, is the cell stiffness influenced by the mechanical properties of its surroundings?

Over the past two decades, several studies supported the idea that cells adapt to the mechanical properties of their environment, including adapting their stiffness [49,50]. However, the discussion is still open and new results could corroborate or modify our understanding of these scenarios.

**Figure 1:**
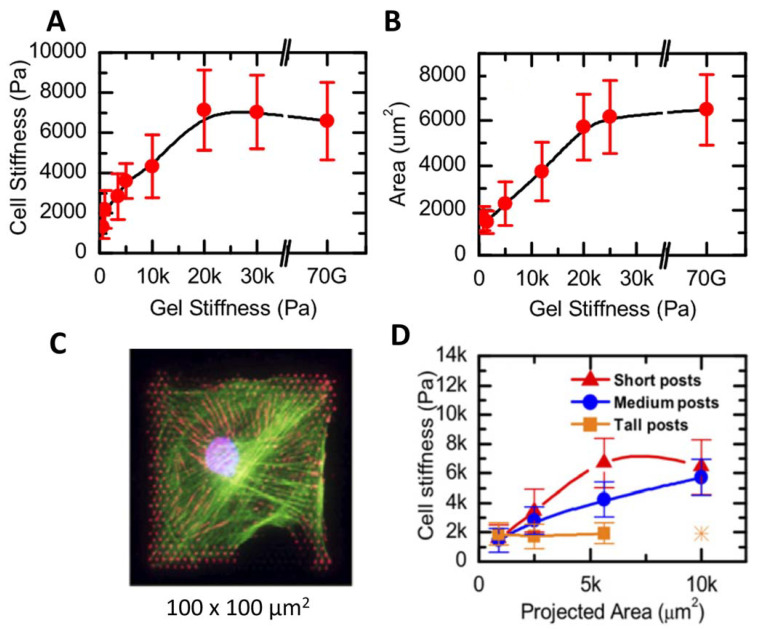
(**A**) Cell stiffness increased as a function of the supporting gel stiffness, reaching a plateau for substrate stiffnesses higher than 20 kPa. (**B**) The same trend was observed plotting the cell area as a function of the substrate stiffness. (**C**) A large number of stress fibers were displayed for spread cells. (**D**) Cell stiffness as a function of cell area. On softer samples, cell stiffness did not depend on the cell area. Reprinted with permission from Ref. [51]. 2011, Biophysical Journal.

To address this point, Tee et al. (2010) [51] developed a model substrate with microfabricated arrays of elastomeric pillars to control the substrate stiffness, following the method proposed by Fu et al. (2010) [52]. The substrate stiffness varied in the range between 1 kPa and 30 kPa; in particular, the stiffness changes inversely with the posts’ length. The Young’s modulus of human mesenchymal stem cells increased as a function of substrate stiffness, reaching a plateau for substrates with an elasticity above 20 kPa (Figure 1A). The increase in stiffness was associated with an increase in cell spreading, quantified by measuring the cell area (Figure 1B). The rise in stiffness and size was associated with the formation of a significant network of stress fibers.

Furthermore, the authors employed microcontact printing [51] to create controlled squared patterns of an adhesion-promoting molecule, i.e., fibronectin, on the microfabricated substrates. The cell growth on these substrates was found to be driven by the presence of fibronectin. The cells had a typical square shape, with different sizes ranging from 30 µm × 30 µm to 100 µm × 100 µm (Figure 1C), resembling the size of the fibronectin spots. The authors probed the cell mechanical response by choosing indentation points far from the nucleus and from the lamellipodia. The stiffness of the cells on tall posts was not influenced by cell shape and size; E was always around 2 kPa. On the contrary, on more rigid substrates the cell stiffness was proportional to the spreading area (Figure 1D).

These results supported the idea that cell mechanics is driven by the mechanical properties of the surrounding environment, and also by the cell shape. An issue that was considered by the authors was related to the patterned structure of the substrates onto which the cells were seeded. The cells’ basal region was either lying on the post surface or freestanding in between two posts. The substrate stiffness varied from 20 MPa on the posts to 0 MPa in the free space. Since the cell elasticity did not display a periodic behavior, the authors concluded that the derived modulus was a good estimation of the mechanical properties of the cells. The elastic moduli of the cells were derived by fitting the force vs. indentation curves using the Sneddon model for a conical indenter [53], following a previously proposed approach [54]. We point attention to the fact that the best approximation for the silicon nitride probes employed in the study should be the Bilodeau formula for a regular pyramidal punch [55], that would maintain the same relation between F, E, and δ, but with some correction factors.

A few years later, a method for quantitative mechanical characterization of soft, heterogeneous samples in 3D was presented [56]. The aim was to demonstrate how to determine the elastic modulus of both a cell and the surrounding ECM by decoupling the force response of the two components.

**Figure 2:**
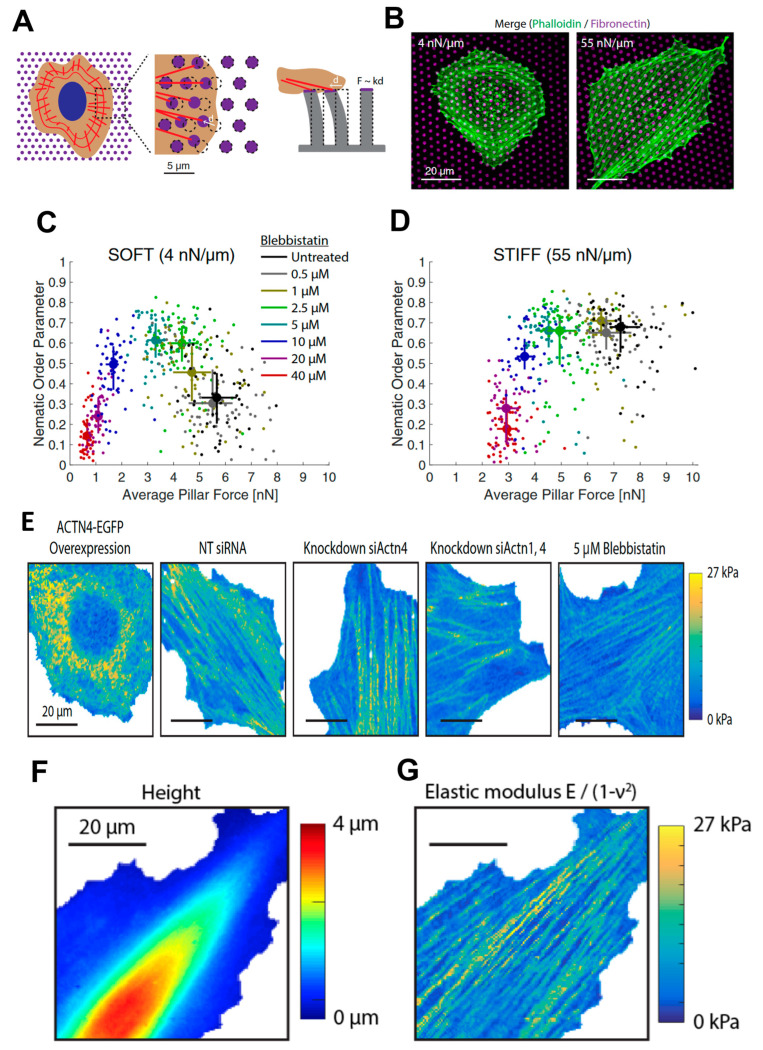
(**A**) Representation of micropillar experiments showing actin (red) and the fibronectin layer on top of the pillars (magenta). (**B**) Cell shape was modulated by substrate stiffness. REF52-WT fibroblasts had a round shape when seeded onto soft (Left) micropillar substrates, while they spread on stiff (Right) micropillar substrates. (**C**) Nematic order parameter versus average traction stress on soft pillars with different concentrations of blebbistatin. Circles and error bars indicate the median ± interquartile range. (**D**) The same result was obtained on stiff posts. Circles and error bars indicate the median ± interquartile range. Data in C and D were merged from two independent experiments per condition. (**E**) Maps of the elastic modulus obtained in QI mode. REF52-Ftractin cells with (left to right) ACTN4-EGFP overexpression, NT siRNA control, ACTN4 knockdown, pan-ACTN knockdown, and 5 μM blebbistatin (scale bars, 20 μm). (**F**) Cell topography was determined from the contact point of each force–indentation curve. (**G**) The elastic modulus of the same cell was determined by fitting each force–indentation curve to the elastic contact model. Reprinted with permission from Ref. [57]. 2022, PNAS.

More recently, Doss et al. [57] thoroughly investigated the cell response to substrate rigidity, focusing on the interplay between the rheological properties of the cytoskeleton and ECM mechanical properties. These mechanisms are at the base of the phenotype differentiation induced by mechanics, a fundamental issue since pathological states can perturb both the environmental stiffness and the cell’s response to the mechanical properties of its surroundings. Doss et al. found that the actin cytoskeleton polarized on stiff substrates but not on soft cells, demonstrating that cells can adopt a rigidity-dependent phenotype. Similar to the previously presented work, arrays of soft pillars were used as substrates for cell culture. In particular, they exploited these devices for a double purpose: to tune the substrate stiffness and to quantify the traction forces exerted by the cells on the substrate (Figure 2A,B). They correlated the traction force with the level of organization of the actin meshwork, characterized by a nematic order parameter [58,59,60] which is zero when actin is isotropic and one when actin is polarized along a certain direction. Treating the sample with blebbistatin, which inhibits myosin II function, they were able to tune both the traction force exerted on the substrate and the cell polarization (Figure 2C,D).

The correlation between cytoskeletal stiffness, actin (ACTN) concentration, and myosin II activity was demonstrated using AFM nanoindentation. In particular, the degree of passive actin (ACTN) cross-linking was controlled with diverse approaches: by using interfering RNA (siRNA)-mediated knockdowns of ACTN1, ACTN4, or both ACTN1 and ACTN4 simultaneously (pan-ACTN), or by transient overexpression of ACTN1-EGFP or ACTN4-EGFP. A control cell line was also treated with blebbistatin that was already used to tune the traction forces. The mechanical properties of the different cell types were assessed by AFM indentation experiments after seeding the cells on standard plastic dishes. A fast force–volume mode, the Quantitative Imaging [61,62,63,64] introduced in 2011, was employed to calculate the local mechanical properties. In particular, 128 × 128 force curves were acquired on a 60 µm × 60 μm area. Each force curve was analyzed using a linearization fitting method of theoretical force–indentation curves from a sphero-conical probe, previously introduced by Staunton et al. [56], and adapted to thin soft samples on infinitely rigid substrates [65]. Testing the mechanical properties of the cell with a lateral resolution of ~500 nm allows the discrimination of different cell compartments. The elastic modulus of the cytoskeleton stress fibers is about an order of magnitude higher than that measured on the other regions of the cell, as shown by the qualitative but clear representation given by the elasticity maps reported in Figure 2E. The elasticity maps confirmed the decrease of stress fiber stiffness after treatment with blebbistatin. In the same work, the authors demonstrated that cell stiffness is proportional to polarization response, showing that in general the lamella region is stiffer than the lamellipodial and nuclear region, the thinner and the thicker parts of the cell, respectively (Figure 2F,G).

Nowadays, the AFM offers the unique capability to provide mechanical analysis with a lateral resolution that allows the discrimination of different cellular compartments.

**Figure 3:**
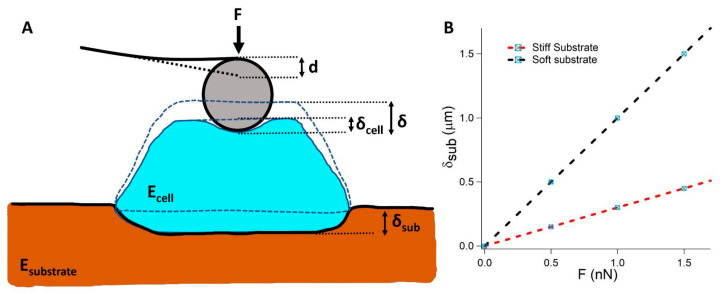
(**A**) Very soft substrates are softer than cells. In this case, when the cell is pushed on the substrates in a force spectroscopy experiment, the cell is acting as an indenter penetrating the soft substrate. (**B**) The indentation increases linearly with the force increase; the slope is higher on softer substrates. Adapted from Ref. [66].

A recently published paper [66] opened an interesting discussion to reconsider the large number of results already published with a critical mind. In particular, the authors focused on very soft substrates, i.e., substrates with a stiffness similar to the cell stiffness or lower, showing that in these cases, when compression is applied to the cell, the substrate deformation is not negligible and must be taken into account in the analysis of AFM-based indentation experiments (Figure 3A,B). The authors proposed a new model in which the cell and the substrate are acting as two springs in series. The Hertz’s contact is used to follow the non-linear behavior of the relationship that describes the force, F, as a function of the indentation, δ. Elastic resonator interference stress microscopy (ERISM) [67] was employed to quantify the vertical deformation of the substrates with high spatial resolution. Both the deformation of the substrate and the stress were highest under the indenter and decreased linearly away from this point. Importantly, both stress and deformation increased linearly with the applied force (Figure 3A). Furthermore, the substrate deformation maintained the same shape at different applied forces (Figure 3B); hence, the contact area was constant at each applied force. This case can be approximated as the indention of an elastic semi-infinite half space by an axisymmetric stress field on a circular region, resembling the case of the indentation by a flat punch. The force–indentation relation is linear, as predicted by the Boussinesq solution.

The two contributions to the total indentation δ:

$$\delta =\delta_{cell}+\delta_{sub}$$

can be expressed as a function of the applied force as:

$$\delta \left(F\right)={\left(\frac{16}{9}E_{cell}\sqrt{R}\right)}^{-\frac{2}{3}\;}F^{\frac{2}{3}}+\frac{9}{4\pi }\frac{1}{RE_{sub}}F$$

This model was named the composite cell–substrate model (CoCS model). The authors pointed out that the model can be applied for other indenter geometries as the tip geometry only affects *δ_cell_*.

Considering the CoCS model, the elastic modulus of the cell is independent of the substrate stiffness. This result suggests a critical analysis of several results that appeared in recent years and opens an interesting discussion in the field of mechanobiology.

## 3. Cell Mechanics and Pathological States

In recent decades, several observations indicated a relationship between some pathological states and cell mechanics. The AFM played an essential role in the characterization of mechanical changes in pathological cells and tissues, and in the investigation of the mechanisms that are responsible for these mutations in cell phenotypes.

### 3.1. Cell Mechanics in Cancer Cells

The more significant part of these AFM studies is focused on cancers. The potential use of AFM to measure cell stiffness as a quantitative biomarker of cancer-related changes has been demonstrated for several cancer types including breast [68,69], prostate [70], ovarian [71], thyroid [72,73], pancreas [74,75], kidney [76], and bladder [77,78] cancers.

The first pioneering study appeared in 1999 when Lekka and co-workers employed a custom-built AFM system to measure the stiffness of different cancer cell lines, compared with non-pathological cell lines [77]. The indentation curves were fitted with the Sneddon model for a conical indenter [53]. The Young’s moduli of cancer cells were about one order of magnitude lower than those of normal cells. The authors proposed that these changes could be associated with an aberrant organization of the filament network of the cytoskeleton. In 2007 Cross et al. [35] reported the first mechanical analysis of ex vivo cancer cells. They examined cells from the body fluids of patients with suspected lung, breast, or pancreas cancer. The choice of cells from body fluids and not from the primary tumor is significant and is particularly suitable for this kind of application. Tumor cells in body fluids are all metastatic. In the samples, both metastatic and benign cells co-existed, i.e., the pathological specimen and the control were simultaneously present. The authors found that cancer cells’ stiffness was significantly lower compared to the stiffness of benign cells. This work also demonstrated that cells from different cancer types have the same mechanical properties. Additionally, in this work, the Sneddon model of contact mechanics was employed [53]. The Sneddon theory considers the sample as a half-plane and the indenter as an axisymmetric solid body. It should be noted that pyramid silicon nitride tips do not satisfy Sneddon’s hypothesis, since they are not an axisymmetric system. On the other hand, we must note that in comparing the stiffness of different cell populations, e.g., malignant vs. benign cells, the choice of the theoretical model is not so critical. The absolute stiffness value varies with the model, but the relative difference in the stiffness of the two populations is maintained.

Palmieri et al. [79] studied colon carcinoma cell lines from the primary tumor and lymph node metastasis. The choice of the cell types followed the same rationale proposed by Cross et al. some years before. Phenotypic variations associated with the metastatic process can be detected by isolating primary and metastatic tumor cells in two distinct populations. Palmieri et al. took the analysis of their experimental data a step further. They considered the non-specific adhesion between cells and AFM tips to refine the Young’s modulus calculation by applying the Johnson–Kendall–Roberts (JKR) model [80]. The mechanical properties of the cells were correlated with the shape, surface roughness, and actin organization. The authors demonstrated that all these physical properties are modulated in cancer cells to favor cell growth and invasion. In particular, they observed that elongated primary tumor cells were characterized by a high stiffness and a low adhesion with the AFM tip, while round-shaped primary cells, which have a decreased doubling time and more tissue-aggressive behavior [81], showed similar tip adhesion values but lower stiffness values. A further decrease in stiffness and an increase in non-specific adhesion was observed for metastatic cells; these features are interpreted as the result of the properties acquired by metastatic cells to survive the shear forces of the lymphatic stream.

In agreement with some conclusions of the study by Palmieri et al., Prabhune et al. [72] found that primary normal thyroid cells are 3- to 5-fold stiffer in comparison to malignant thyroid cells. The authors correlated their results to the duration between cell seeding and AFM experiments and suggested that the differences observed in the actin organization between malignant and normal thyroid cells directly contribute to the alteration of cell mechanics in cancer cells. In another study from the same group, Rianna et al. [73] studied the response of normal and cancer renal cells to changes in microenvironment stiffness and topography. The authors used two materials, polyacrylamide (PA) and polydimethylsiloxane (PDMS) as cell culture supports mimicking, respectively, the stiffness values previously measured in vivo in human kidney tissues by using magnetic resonance elastography and the microenvironment topography that cells may encounter in tissues. An interesting observation from their study was that metastatic cancer cells were stiffer than their normal counterparts when seeded on soft gels. The opposite behavior was found when the cells were seeded on stiffer gels and conventional culture systems, in agreement with the common assumption that cancer cells are softer than their healthy counterparts. A related aspect was investigated by Abidine et al. [78]. The authors found that when seeding cancer cells on an endothelial monolayer (HUVEC cell monolayer substrate), the cells did not spread and maintained a round shape, and showed stiffer elasticity values. The opposite trend was found on polyacrylamide gels with the same stiffness as the HUVEC substrate (8 kPa). The authors correlated this stiffening of invasive cancer cells on HUVEC substrates with the actin reorganization during transmigration. In a recent work, Stylianou et al. [75] observed the effect of collagen stiffness on pancreatic fibroblasts and cancer-associated fibroblasts (CAFs). They demonstrated that the collagen stiffness altered the invasion properties of fibroblasts and cancer-associated fibroblasts by modulating cytoskeleton remodeling.

### 3.2. Cell Mechanics on Neurodegenerative Diseases

**Figure 4:**
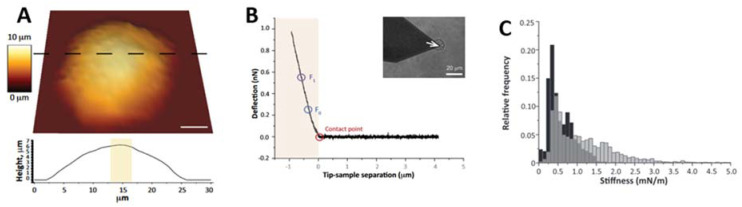
(**A**) A low-resolution topography of the nucleus can be obtained from F-D curve analysis. Nucleus thickness is derived from AFM topography. The 40 × 40 F-D curves (n = 1600) were acquired from a 30 μm × 30 μm square area. (**B**) A typical F-D curve acquired from an isolated nucleus. Nuclear stiffness was derived from all the acquired F-D curves, calculating the slope of the linear fit performed between two points at fixed forces, F_0_ = 0.25 nN and F_1_ = 0.55 nN. Inset: AFM probe positioned above an isolated nucleus. A spherical indenter was positioned at the cantilever free end (white arrowhead). (**C**) Nuclear stiffness increased in ADLD human skin fibroblasts as can be seen in the representative stiffness distributions accumulated from CTR (black) and ADLD (gray) cells. Reprinted with permission from Ref. [90]. 2014, The FASEB Journal.

Although most of the AFM studies on pathologically induced modifications of cell mechanics are related to cancers, a small number of works focused on other disorders, such as neurodegenerative diseases. In fact, AFM has been used to evaluate the mechanobiology of the brain in vitro (for review see [82,83,84]). In particular, the technique was used to evaluate the shift in stiffness in developing brain tissue [85], demonstrating that the mechanical behavior of single, dissociated cells cannot explain the tissue-level stiffness, which is also affected by the extracellular matrix and by tissue structure. A similar result was obtained by Nagasaka et al. [86] when studying the mechanical properties of the developing cerebral cortical proliferative zone in mice and ferrets, both at the tissue and single-cell level. In 2000, Bhatia et al. [87] applied AFM imaging to demonstrate that morphological changes observed in aged human fibroblasts were a common indicator of cellular degeneration depending on the alloform of induced Aβ oligomers. Lulevich et al. [88] introduced a single-cell compression method where a large colloidal probe is used as the indenter to compress the cell, mimicking the compression of a single cell between two parallel plates. This method provides a good estimation of the osmotic pressure and variation in intracellular ion concentrations. When this method was applied to N2a neuroblastoma and HT22 hippocampal neuronal cells, it was found that the Aβ(1-42) treatment induced a membrane elasticity stiffening for both cell lines, likely related to the insertion of the amyloid oligomers into the plasma membrane. On the other hand, Ungureanu et al. performed measurements after inducing smaller cell deformations to investigate the influence of Aβ oligomers in neuronal elasticity [89]. To this end, the authors used polystyrene beads with a diameter of 15 μm as the indenter, applying forces below 1 nN. The authors employed hippocampal neurons from rat embryos at three different ageing stages and observed that the Aβ(1-42) treatment induced a decrease in elasticity of primary neurons which was related to the decrease in the membrane cholesterol level due to ageing. The treatment with Aβ(1-40) resulted in an increase in 21 DIV neurons which the authors suggested could be promoted by the absorption of protein aggregates at the cell surfaces. The main difference between these two works is the force regime used by the authors. While Lulevich et al. applied high forces with a large indenter, inducing large cell deformations and using the osmotic pressure as the main outcome of the AFM measurement, Ungureanu et al. utilized a more sensitive regime, inducing small cell deformations and focusing on the elastic response provided by the plasma membrane and the components that are closely associated with it, i.e., the actin cortex. It is with the latter regime that Ferrera et al. [90] studied the mechanical properties of cell nuclei from patients with autosomal dominant leukodystrophy (ADLD). This pathology is associated with the overexpression of lamin B1, a protein of the nuclear lamina. The nuclear lamina has a fundamental role in the determination of the nuclear shape and mechanical properties. The characterization of nuclear mechanics by AFM is particularly challenging, since AFM is a contact technique and the presence of the cell cortex surrounding the nucleus affects AFM indentation experiments. The AFM tip is directly in contact with the cell membrane and the presence of the rigid cytoskeleton does not allow access to the nuclear stiffness. Osmotic disruption was employed to extract the nucleus from the cell, enabling the direct contact between the AFM probe and the nuclear envelope, i.e., the subject of the investigation. The authors investigated the mechanical properties of nuclei isolated from pathological cells and healthy controls. A typical topographical reconstruction of an isolated nucleus analyzed by Ferrera et al. is reported in Figure 4A. The indentation curves acquired from nuclei significantly deviated from the typical behavior for elastic materials, showing an almost linear relationship between the force and indentation. For this reason, the authors considered the slope of the curves between two fixed force values (F0 and F1 in Figure 4B,C) as an indicator of the nuclei stiffness. This method, proposed in a previous work [91], aimed to avoid systematic errors induced by the incorrect choice of theoretical model. The stiffness values derived from this method did not unequivocally describe the mechanical properties of the sample, since the slope of the curve depends on the indenter shape, a geometrical parameter that, in this case, was neglected. The pathological nuclei were found to be stiffer than nuclei from healthy cells, likely due to the higher concentration of lamin B1 in the nuclear lamina. The Young’s modulus was also calculated using the Hertz model for a spherical indenter. The elastic moduli of nuclei from ADLD patients were higher than those of control nuclei. These results confirmed that, although the application of a correct theoretical model is necessary to determine the effective elastic modulus of a sample, it is still possible to highlight differences between classes of samples even when applying less refined, non-ideal models. In general, although most of the works on cell mechanics focus on elastomechanical properties, deeper analyses demonstrated that living cells behave as a complex active material with both solid-like elastic and fluid-like viscous properties [92,93]. Exploiting the dependence of the mechanical properties of cells on the experimental timescale, an approach based on the application of step and cyclic loads can provide the power-law rheology and the unified relationship between the power-law exponent and cell stiffness [94], opening new perspectives in the study of the modifications induced by pathological states.

## 4. Steps towards the Development of New Diagnostic Tools: The Use of AFM on Tissues

**Figure 5:**
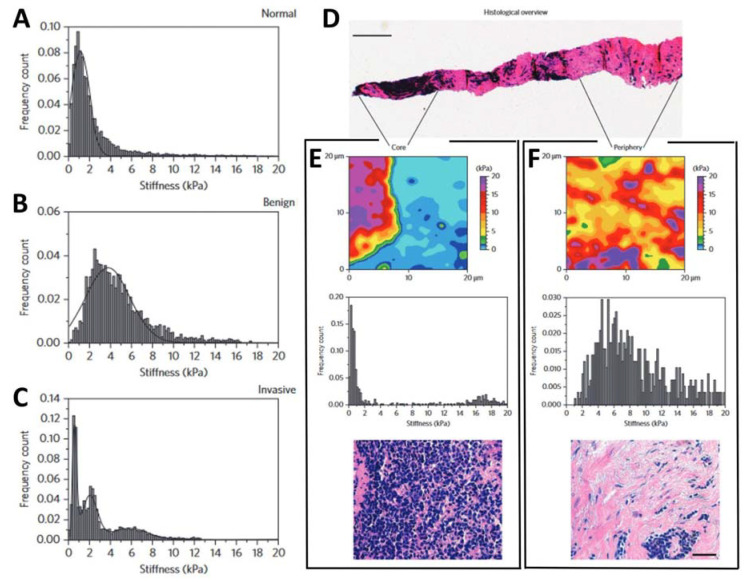
(**A**) The stiffness distribution for healthy mammary gland tissue was unimodal. (**B**) The analysis of the entire benign lesion revealed a unimodal but broader stiffness distribution. The stiffness increased compared with the healthy biopsy. (**C**) The stiffness distribution from a malignant tumor was multimodal. (**D**) The stiffness of cancer biopsies was different from the core to the periphery of the biopsies. Overview of the entire cancer biopsy with reference to the areas mapped in detail (scale bar, 500 µm). (**E**) Top: representative AFM stiffness map 24 pixels × 24 pixels of the core region. Middle: the stiffness distribution exhibited pronounced softness within a narrow peak. Bottom: a dense population of cancer cells was located at the core region of the local histology. (**F**) Top: the stiffness map 24 pixels × 24 pixels at the periphery of the histological sample demonstrated stiff features. Middle: the stiffness distribution was broader and shifted towards higher values. Bottom: the periphery of the sample was mainly composed of fibrotic tissue. Scale bar, 50 µm (also applies to the image in E, bottom). Reprinted with permission from Ref. [95]. 2012, Nat. Nanotechnol.

A step towards the development of an AFM-based system for cancer diagnosis is represented by its direct application on human tissues. Standard diagnostic methods are based on immunohistochemical analysis of biopsies, i.e., part of tissues isolated from patients’ organs. This method is well-established and provides effective results. On the other hand, in some cases, the low specificity and sensitivity represent a limit for early diagnosis, generating a not negligible number of false negatives. For this reason, the development of new tumor markers is fundamental to increasing the efficiency of cancer therapies. The study of 30 human breast biopsies carried out by Plodinec et al. [95] demonstrated that biopsies from malignant breast cancer have a typical nanomechanical signature, detectable by AFM nanoindentation. In particular, pathological samples have a broad and multimodal stiffness distribution, deriving from the high tissue heterogeneity induced by pathological modifications, while healthy and benign fibroadenoma cells have a unimodal stiffness distribution. The study reports that healthy cells have an average Young’s modulus of 1.13 kPa ± 0.78 kPa (Figure 5A) while benign fibroadenoma cells have a higher stiffness of 3.68 kPa ± 1.92 kPa (Figure 5B), likely due to the typical high fibrotic content. Measurements on cancer biopsies provide multimodal stiffness distributions (Figure 5C). In particular, the presence of a significant peak at low stiffness 0.57 kPa ± 0.16 kPa was associated with the decreased rigidity of cancer cells, demonstrated previously for different kinds of cancer cells in single cell studies [35]. The presence of two other distinct peaks indicated the marked mechanical heterogeneity across the pathological sample.

By performing measurements on defined regions of the biopsy (Figure 5D), the core (Figure 5E) and the periphery (Figure 5F), and comparing the mechanical and histological data, the authors were able to highlight not only pathological mechanisms that alter the physiological properties of cells and tissue, but also to exploit it as a biomarker for diagnostic and prognostic purposes. The authors also claimed that the high-resolution stiffness mapping allowed them to reveal that cancer progression is associated with a significant softening of tumor epithelial cells in comparison to normal mammary epithelium and is not limited to matrix stiffening, as previously assumed.

The measurement of the elastic modulus has also been used for the determination of the normal and pathological ranges of matrix stiffness of liver tissue as a diagnostic biomarker for pathological staging of hepatic fibrosis [96,97,98,99]. The stiffening of the normal tissue liver matrix to higher values in fibrotic tissue was directly correlated to the modulation of various primary hepatocytes functions [96] and to the extent of fat accumulation on hepatocytes [98]. In a recent study, Tian et al. [99] demonstrated that indentation-type atomic force microscopy can be used to correlate changes in nanomechanical properties during liver cancer progression. In fact, the authors, like Plodinec et al., found a unimodal trend for healthy tissues that became more heterogeneous with the progression of the tumor. The characteristic low elasticity peak (LEP) centered at around 1–1.5 kPa for healthy tissues was found to be lower in HCC and recurrent HCC tissues, while no significant differences were observed for cirrhotic tissues. The latter, however, presented two or more peaks in its elasticity distribution. Interestingly, the high elasticity peak (HEP) observed around 2 kPa for paraneoplastic tissues was associated by the authors to chronic hepatitis B virus (HBV) infection indicating that the patient history must be taken into consideration while evaluating the nanomechanical properties of tissues for diagnostic purposes.

In the following years, other works investigating the stiffness of tumor tissues have been presented by the same and other groups [100,101,102,103]. These results drove the development of the first commercial nanomechanical tool for cancer cell diagnosis, translating the results of years of research into a commercial tool for biomedical applications.

## 5. Single-Cell Force Spectroscopy to Address Cell Adhesion Changes in Pathologies

Cell adhesion drives the organization of cells in multicellular organisms, playing a crucial role in physiological functions that regulate the formation and development of tissues. Cell adhesion is also a primary issue in modern tissue engineering [104]. Several works demonstrated that cell adhesion capability is impaired in several pathological states, such as cancer and multiple sclerosis.

These motivations stimulated the development of new experimental approaches for the quantitative characterization of cell adhesion properties, including single-cell force spectroscopy (SCFS) [105,106,107], which has led to extraordinary progress in the field.

The idea of SCFS is to substitute the AFM tip with a living cell as a probe. The use of a complex, live, and inherently multifunctional probe improved the capability of AFM force spectroscopy.

Here, we review the essential goals of SCFS, starting from the studies of pathological mechanisms, and finally presenting the improvements in tissue engineering and other technological fields.

### 5.1. Cancer Cell and Cell Adhesion

Metastatic breast cancer cells (BCa) can colonize the skeleton. Taubenberger et al. studied the mechanism that regulates BCa cell migration [108]. Matrices secreted by human primary osteoblasts (hOBMs) isolated from human trabecular bone [109] simulated the bone microenvironment. hOBM matrices contained typical bone proteins, such as collagen type I, fibronectin, osteonectin, and osteocalcin, presenting a dense fibrillary meshwork that resembles the native trabecular bone. Four different BCa cell lines with different metastatic potentials were cultured on substrates coated with hOBM matrices. In particular, three metastatic cell lines, SUM1315, MDA-MB-231, and MDA-MB-231BO, were used. A nonmetastatic BCa cell line with a luminal phenotype was employed as a control.

**Figure 6:**
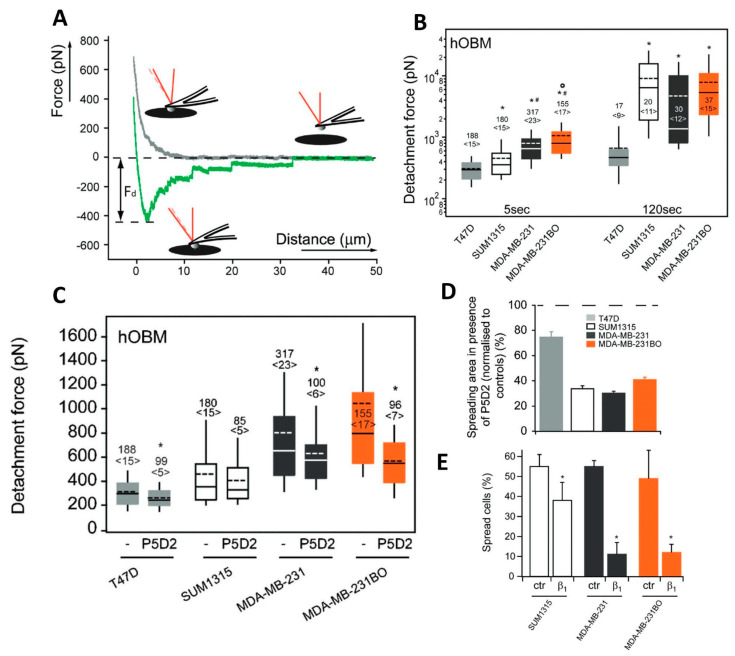
(**A**) Representation of the cell adhesion experiment. The grey line is the approach part of the F-D curve, while the green one is the retraction part. Starting far above the sample (right part of the curve, grey line) the cell was moved downwards and pushed on the substrate. After a defined contact time, the cell was retracted (green line), and the detachment from the adhesive substrate (hOBM or COL I) was registered. F_d_ is the detachment force, i.e., the maximum traction force reached before the detachment process started. (**B**) Detachment forces of BCa cells on hOBM for contact times of 5 s and 120 s. Numbers indicate the total of analyzed F-D curves, the number of probed cells is indicated in parentheses. Top and bottom of the boxes indicate the 75th and 25th percentiles, and upper and lower whiskers the 90th and 10th percentiles. Solid lines within boxes denote the median, and dotted lines denote mean detachment forces. Statistical differences among cells were verified using Kruskal-Wallis tests. Contrasts were calculated for the individual pairs. Significant differences (*p* < 0.05) among pairs are indicated as follows: *, any-T47D; #, any–SUM1315; °, MDA-MB-231–MDA-MB-231BO. (**C**) Detachment forces of BCa cells on hOBM in presence of 6 μg/mL β1-integrin function-blocking antibody (P5D2). Top and bottom of the boxes indicate the 75th and 25th percentiles, and upper and lower whiskers the 90th and 10th percentiles. Solid lines within boxes denote median, and dotted lines denote mean detachment forces. Results obtained in the presence or absence of the blocking antibody were compared employing a nonparametric Mann-Whitney test. Significant differences (*p* < 0.05) among pairs are indicated by *. Numbers of analyzed curves/probed cells are given as in (**B**). (**D**) Mean spreading area (average ± standard deviation) of cells treated with P5D2 normalized to control cells. At least 90 cells were considered for each cell type and condition. (**E**) Spreading of β1-integrin knockdown cells and control cells (percentage of spread cells) evaluated after 6 h of incubation on hOBM. Data sets of control and β1-integrin knockdown cells were compared using a *t* test. Significant differences (*p* < 0.05) among pairs are indicated by *. Reprinted with permission from Ref. [108]. 2013, J. Bone Miner. Res.

BCa cells tend to polarize on hOBM-coated substrates, to an extent proportional to the metastatic potential. On the contrary, all the cell types maintained an isotropic shape on standard adhesion-promoting substrates. SCFS was employed to verify whether the shape assumed by cells on hOBM is associated with different adhesion properties. Single cells from the four different cell lines were attached to AFM tipless cantilevers, exploiting a multistep functionalization method that had been previously proposed [105]. The cells were kept in contact for 5 s and 120 s with both hOBM- and collagen I (COLI)-coated substrates. The cell adhesion properties were quantified considering the detachment force, that is the maximal traction force registered (Fd, Figure 6A). After 5 s of contact, the detachment force increased significantly for cells with a higher metastatic potential. For longer contact times (120 s), a remarkable increase in the detachment force (20-fold) for malignant cells was observed, while a slight change was registered for the control cells (Figure 6B). In contrast, on COL I the detachment force of malignant cells also did not significantly increase.

A deeper analysis was oriented toward the definition of the main molecular species involved in the adhesion of control and malignant cells. BCa cell surface and gene expression levels of the main integrin species were used to define the expression pattern of the different cell lines.The adhesion force measured on COL I was proportional to the expression level of α2-integrin, that, on the contrary, did not influence the adhesion on hOBM. This evidence suggested that α2-integrin mediates cell adhesion to COL I, but not on hOBM. The detachment forces on hOBM increased as a function of the expression of β1-integrin. The role of β1-integrin in BCa adhesion to hOBM was investigated further. The cells were treated with P5D2, a specific antibody that blocks the function of β1-integrin, before the adhesion measurements (Figure 6C). The adhesion on hOBM decreased for all the cell lines, although to different extents. Furthermore, treatment with P5D2 inhibited the growth and spreading of cells on hOBM (Figure 6D), and knockdown of β1-integrin significantly affected the spreading capability of malignant cells (Figure 6E). All these findings indicate that β1-integrin regulates the adhesion of BCa cells to hOBM. Other integrins were tested following the same methodology. The negative results indicated that they were not involved in BCa adhesion mechanisms, confirming that BCa cell adhesion to hOBM is mediated by β1-integrins.

A few years later, Smolyakov et al. [110] exploited the SCFS approach to provide a more complete characterization of cancer cells, focusing on four breast cancer cell lines with different invasive potentials. Through SCFS, they studied the adhesion of the cancer cell lines on fibronectin and on other cells. The experimental method was similar to that applied by Taubenberger et al., but a deeper analysis of the force curves was employed to extract information not only on cell adhesion but also on cell elasticity and on the tendency to form membrane tethers.

### 5.2. Misfolded Protein Aggregates and Cell Adhesion

**Figure 7:**
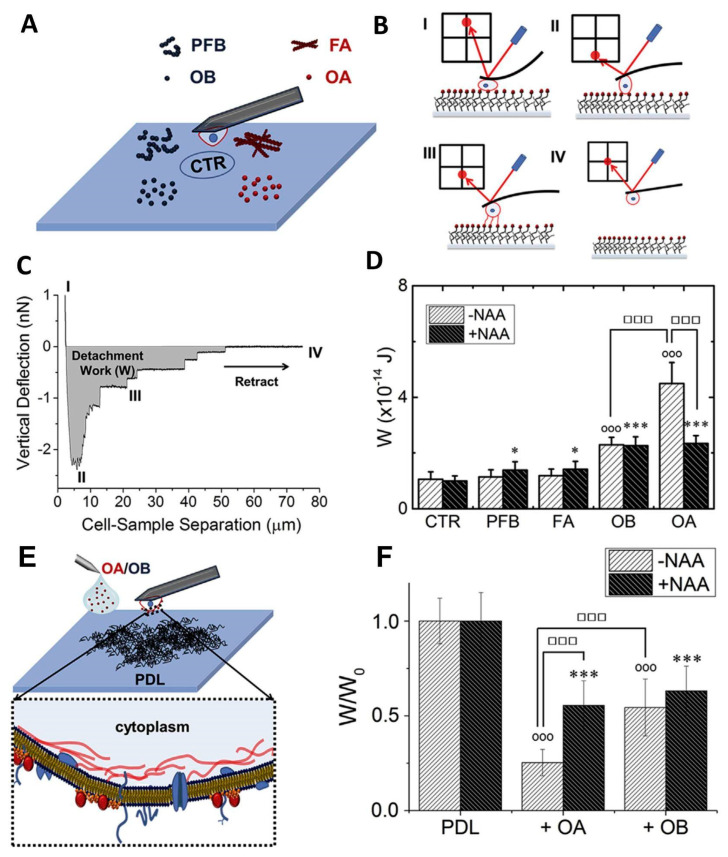
(**A**) Schematic representation of the experimental setup with OA, OB, FA, and PFB aggregates on different regions of the functionalized glass substrate. (**B**) Cartoon of the four steps of the experiment. First, the cell was in contact with the functionalized substrate under a defined force (I); then, the AFM cantilever moved away from the substrate until the maximal adhesion force was reached. At this time, no adhesion interactions have been broken and the cantilever was applying a traction force on the cell (II). The cell was then gradually detached from the substrate; the detachment process was characterized by several rupture events, corresponding to jumps of the traction force (III). A final plateau at a force of 0 nN was reached when the cell was fully detached (IV). (**C**) Typical F–D curve acquired using SCFS with the four steps described in (**B**). The detachment work (W), represented by the gray area below the baseline at a force of 0 nN, quantifies the interaction between the cell and the substrate. (**D**) Mean work spent to detach the cell from the substrate (W) for the various aggregate types and obtained from cells treated and not treated with NAA. Error bars correspond to standard deviations. * and *** indicate *p* ≤ 0.05 and *p* ≤ 0.001, respectively, relative to CTR. °°° indicates *p* ≤ 0.001 relative to CTR in the experiments on cells not treated with NAA. ^☐☐☐^ indicates *p* ≤ 0.001 in the indicated comparisons. (**E**) Schematic representation of the SCFS experiment to test CHO cell adhesion on PDL in the absence and presence of OA or OB. (**F**) Mean work spent to detach the cell from PDL-coated substrates obtained in the absence (PDL) and in the presence of OA/OB (+OA/+OB). Results obtained from NAA-treated cells are represented by the black bars. All the results are normalized to the corresponding value obtained in the absence of protein aggregates, here indicated as W0. Error bars correspond to standard deviations. *** and °°° indicate *p* ≤ 0.001 relative to PDL. ^☐☐☐^ indicates *p* ≤ 0.001 in the indicated comparisons. Reproduced Reprinted with permission from Ref. [111]. 2018, Biophysical Journal.

SCFS can also provide valuable results in studying mechanisms that, at first glance, are not considered directly related to cell adhesion. The interaction of the cell membrane with external agents, such as protein aggregates, viruses, or nanoparticles, can determine the cell’s fate. These phenomena are often characterized using model systems (liposomes, supported lipid bilayers). Proposing a tailor-made experimental procedure, SCFS has been employed to study the interaction between amyloid oligomers and cell membranes [111]. The study was conducted on HypF-N aggregates. HypF-N is the N-terminal domain of HypF, a bacterial protein that is not related to protein misfolding diseases in vivo. Despite this, HypF-N can form amyloid aggregates in vitro. It has been demonstrated that the toxicity of HypF-N oligomers depends on the aggregation conditions. In particular, aggregation in the presence of trifluoroethanol produces oligomers, indicated as OA by Oropesa-Nuñez et al. [111], showing a toxicity pathway that resembles that of oligomers from Aβ peptides. On the contrary, aggregation in acidic conditions gives rise to non-toxic oligomers called OB. Larger fibrillar aggregates obtained in both conditions are non-toxic. Although HypF-N aggregates can be considered a model system, they have contributed significantly to the understanding of the interplay between oligomer structure and toxicity [112,113], as well as to the characterization of the interaction between misfolded protein oligomers and model lipid membranes [114]. Oligomer-membrane interactions are considered the earliest event in oligomer-mediated cytotoxicity. In particular, it was shown that GM1, a negatively charged glycolipid, triggers OA toxicity. Furthermore, OA were able to bind to the ordered phase of the model lipid membrane only in the presence of the negatively charged GM1. SCFS allows for the testing of the binding capability of OA on “real” cell membranes. Chinese Hamster Ovarian (CHO) cells, used as a probe for this purpose, were attached to a tipless AFM cantilever using a previously proposed method [115]. Four types of HypF-N aggregates were covalently bound to a glass coverslip: OA, OB, and the fibrillar forms FA and PFB obtained in trifluoroethanol and acidic conditions, respectively (Figure 7A). The cell binding capability was assessed by pushing the cell on the coverslip with a controlled force of 1 nN and retracting the cell after a defined time period of 30 s (Figure 7B). The detachment work (W) was used to quantify the affinity between cells and aggregates (Figure 7C). The experiments demonstrated that cell binding was significantly higher for toxic OA aggregates than for the harmless OB (Figure 7D). The cells did not interact with fibrillar aggregates. Notably, repeating the experiment after cell treatment with a neuraminidase cocktail (NAA), i.e., enzymes that remove the negative charge from GM1 by cutting the sialic acid group, the adhesion to OA and OB was comparable (Figure 7D). This result related the toxicity of aggregates to their capability to bind the plasma membranes, indicating that GM1 is the mediator for oligomer recruitment. Furthermore, single-cell adhesion measurements were performed on poly-D-lysine (PDL)-coated substrates in the presence and in the absence of OA or OB (Figure 7E). A previous work showed that CHO adhesion on PDL was mainly driven by RGD-binding integrins [116]. The presence of oligomers in solution resulted in a decrease of the adhesion strength on PDL, and in this case, the effect of OA was higher than that of OB (Figure 7F). Cell adhesion capability was impaired by the presence of toxic oligomers, suggesting an influence of OA on RGD-binding integrin activity. Additionally, in this case, the removal of the sialic acid group influenced the action of OA; after NAA treatment, OA and OB modified CHO adhesion on PDL to the same extent. This work confirmed the importance of GM1 in the toxicity cascade of amyloid aggregates, a phenomenon that starts with the recruiting of oligomers at the cell membrane. Furthermore, the authors showed, for the first time, an effect of the oligomers’ presence on cell adhesion due to a failure of RGD-binding integrin function. The decrease in RGD-binding integrin activity is not due to the direct interaction between oligomers and the adhesion molecules but is mediated by GM1, which was confirmed as the main recruiter of misfolded HypF-N oligomers at the cell membrane.

The main papers analyzed in this review are summarized in Table 1.

## 6. Conclusions

Since its introduction in 1986, the AFM has been employed in the study of biological materials. The high-resolution imaging capability of the AFM cannot be fully exploited in complex systems such as biological cells. Despite this, the use of the AFM in the mechanobiology field has been constantly increasing and nowadays the AFM is considered the gold standard method in the characterization of cell mechanics. The refinements of the experimental setups and the optimization of the data processing procedures make modern setups readily available for biological and biomedical investigations. AFM investigations shed light on many molecular mechanisms related to pathological states. In recent years, changes in cell mechanical properties have been considered a biomarker of pathological conditions such as tumors, paving the way for the exploitation of the AFM as an advanced diagnostic tool. Like all the techniques, the AFM suffers some limitations. The AFM is a surface technique and therefore it cannot measure the mechanical properties of the inner part of a thick sample, being limited to its upper layer. Furthermore, the AFM does not provide any chemical clues, being unable to directly associate any mechanical change to a difference in sample composition. For this reason, the development of integrated systems coupling the AFM with other instruments, such as with fluorescence microscopy, Raman spectroscopy, or electron microscopy, is a crucial step in the evolution of the AFM technique and will greatly contribute to increasing its field of applications. In mechanobiology, the correlation of the AFM force analysis with the chemical recognition provided by fluorescence [56,63,117,118,119] or Raman [120] signals significantly improve the effectiveness of mechanical investigations. Modern AFM systems can provide a local mechanical characterization with a lateral nanometer resolution. Thanks to correlative microscopy, it would be possible to associate the mechanical changes to local compositional variations with details that, in the case of super-resolution techniques, can overcome the diffraction limit.

## Figures and Tables

**Table 1:** Summary of the main papers analyzed in this review.

	Type of Sample	Main Findings
Tee et al. (2011) [51]	Human mesenchymal stem cells	Cell stiffness and spreading depend on substrate stiffness.
Doss et al. (2020) [57]	Fibroblast cells	Correlation between cytoskeletal stiffness, actin concentration, and myosin II activity.
Rheinlanender (2020) [66]	Microglial cells	Tip indentation depends on substrate deformation. Cell stiffness on very soft substrates is underestimated.
Lekka (1999) [77]	Malignant and benign cells	Stiffness variability between malignant and benign cells.
Palmieri (2015) [79]	Colon Carcinoma cell lines from the primary tumor and lymph node metastasis	Correlation between mechanical properties and cell characteristics (using Johnson–Kendall–Roberts model).
Prabhune (2012) [72]	Primary normal and malignant thyroid cells	Stiffness comparison between normal and malignant cell population.
Rianna (2017) [73]	Normal and cancer renal cells	Normal and cancer renal cell response to stiffness and microenvironment topography changes.
Abidine (2018) [78]	Epithelial bladder cancer cells	Morphological and stiffness variation of cancer cells depends on substrate.
Stylianou (2019) [75]	Pancreatic fibroblasts and cancer-associated fibroblasts	Effects of collagen stiffness on different cell populations.
Bhatia (2000) [87]	Aged human fibroblasts	Morphological changes in aged human fibroblasts as indicator of cellular degeneration.
Lulevich (2010) [88]	Neural cells	Estimation of osmotic pressure and variation of intracellular ion concentrations.
Ungureanu (2016) [89]	Hippocampal neurons from rat embryos	Influence of Aβ oligomers in neuronal elasticity.
Ferrera (2014) [90]	Human skin fibroblasts	Mechanical properties of cell nuclei from patients with Autosomal Dominant Leukodystrophy (ADLD).
Plodinec (2012) [95]	Human cancer biopsies	Study of pathological mechanisms that alter physiological properties of cells and tissues.
Taubenberger (2013) [108]	Metastatic cell lines: SUM1315, MDA-MB-231, and MDA-MB-231BO	Quantification and differentiation of cell-dependent adhesion properties.
Smolyakov (2016) [110]	Breast cancer cell lines with different invasive potentials	SCFS study of cancer cell line adhesion on fibronectin and on other cells.
Oropesa (2018) [111]	CHO cells	HypF-N toxic oligomers bind to the plasma membrane and affect cell adhesion capability.

## Data Availability

Not applicable.
